# Multilayered Graphene Nano-Film for Controlled Protein Delivery by Desired Electro-Stimuli

**DOI:** 10.1038/srep17631

**Published:** 2015-12-01

**Authors:** Moonhyun Choi, Kyung-Geun Kim, Jiwoong Heo, Hyejoong Jeong, Sung Yeol Kim, Jinkee Hong

**Affiliations:** 1 School of Chemical Engineering & Materials Science, Chung-Ang University, Seoul 156-756, Republic of Korea; 2 School of Mechanical Engineering, Kyungpook National University, Daegu 702-701, Republic of Korea

## Abstract

Recent research has highlighted the potential use of “smart” films, such as graphene sheets, that would allow for the controlled release of a variety of therapeutic drugs. Taking full advantage of these versatile conducting sheets, we investigated the novel concept of applying graphene oxide (GO) and reduced graphene oxide (rGO) materials as both barrier and conducting layers that afford controlled entrapment and release of any molecules of interest. We fabricated multilayered nanofilm architectures using a hydrolytically degradable cationic poly(β-amino ester) (PAE), a model protein antigen, ovalbumin (OVA) as a building block along with the GO and rGO. We successfully showed that these multilayer films are capable of blocking the initial burst release of OVA, and they can be triggered to precisely control the release upon the application of electrochemical potential. This new drug delivery platform will find its usefulness in various transdermal drug delivery devices where on-demand control of drug release from the surface is necessary.

On-demand release of therapeutic molecules is essential in various biomedical fields including drug delivery, tissue engineering, biomedical devices, and *in-vitro* diagnostics^1^,^2^,^3^,^4^,^5^,^6^,^7^. While the delivery of an adequate dosage within specific time frame can be achieved using various different drug delivery systems (e.g. vaccination device), it is still challenging to adjust the release profile precisely, especially when the patient’s needs change over the course of treatment^8^,^9^,^10^,^11^.

Recently, active drug delivery systems have been developed that allow for further control over the release of therapeutic molecules^12^,^13^,^14^. This delivery can be triggered and controlled by heat, ultrasonics, near infrared (NIR), and electrical stimulation^15^,^16^,^17^. However, these systems still require judicious selection of materials and structures suitable for the stimulation applied, high drug loading, and precise control over the release of the therapeutic molecules. Cui *et al.* successfully demonstrated an electrically controlled release of the anti-inflammatory drug dexamethasone using a conducting polymer(CP) and graphene oxide(GO)^18^. The conductive network was formed by CP, and the volume change upon application of electrical potential controlled drug release kinetics. The high surface area of GO improves drug loading.

The layer-by-layer (LbL) technique is a versatile means for fabricating nanostructured films composed of multilayers of drugs and other components of opposing charge, in order to induce passive controlled release of the drugs^19^,^20^,^21^,^22^,^23^,^24^,^25^. Thickness, structure, and composition can be easily controlled so that the delivery is pre-programmed into the layered structure of the films, which can change in response to pH, environmental stimulus, or erosion^26^,^27^,^28^,^29^,^30^. These LbL multilayer films have advantages over other drug delivery systems because multilayers could be designed to release multiple components and separately regulate the kinetics of release of individual drug components to optimize a therapeutic response. This versatility of multilayer assembly would find its usefulness in a variety of surface delivery settings, including coatings of simple skin adhesive devices^14^,^31^. Recently, multilayered GO employed in LbL films has been shown to prevent the burst release of a protein, a detrimental characteristic common to many other drug delivery systems^31^,^32^. This was attributed to the GO layers serving as a barrier that prevented interdiffusion of molecules between the layers. Although these previously reported LbL films are promising, they have less flexibility in terms of on-demand controlled release, because the pre-programmed release kinetics cannot be adjusted during administration.

In the present study, we demonstrate an active, on-demand model vaccine antigen delivery system based on LbL nanofilms with multilayers of reduced graphene oxide (rGO) and GO, and enabled by electrical stimulation (Fig. 1). The rGO layers are included in the film for increased conduction upon the application of electrical stimulation, because although GO can serve as a barrier layer for sustained protein release, its low conductivity is not suitable for the facile transfer of electrons in electrical stimulation. Therefore, we incorporated rGO, whose conductivity is typically a few orders of magnitude higher than GO^33^. We used ovalbumin(OVA), a 45 kDa globular protein used as a model vaccine antigen^34^,^35^,^36^,^37^. Our LbL film was demonstrated to be capable of both sustained and real-time control of therapeutic molecule release. To the best of our knowledge, this is the first report demonstrating on-demand protein release from LbL films using electrical stimulation and rGO/GO composites.

## Methods

### Materials

Poly(β-amino ester) (PAE) (M_n_: 17,500) were synthesized as previously described^38^ (supporting information S1). Fluorescein-conjugated ovalbumin (OVA) were purchased from Invitrogen (Eugene, OR).

### Preparation of GO Suspension

Graphite oxide (GO) was synthesized from graphite powder (45 micron, Sigma–Aldrich) using the modified Hummers method and was reduced according to previously described procedures^39^,^40^. Carboxylic acid groups (COOH) were introduced to produce the negatively charged GO (GO−COO−). Positively charged GO sheets were prepared by introducing amine groups (NH_2_) to the surface of the negatively charged GO sheets through a N-ethyl-N′-(3-dimethyl-aminopropyl)carbodiimide methiodide (EDC)-mediated reaction between the carboxylic acids (and/or epoxides) and excess ethylenediamine. This resulted in a positively charged stable GO suspension (GO−NH_3_^+^).

### Film Preparation

All LbL films were assembled with a customized programmable in-house machine. The films were constructed onto an FTO-coated glass substrate that was subjected to O_2_ plasma treatment for 5 min prior to use. The substrate was then immersed in PAE solution (2.0 mg/mL in 100 mM NaOAc buffer) for 10 min, followed by three sequential rinsing steps with pH-adjusted MilliQ water for 1 min each. We then prepared the (r)GO−COO/(r)GO−NH_3_+ layers. The substrates were first immersed for 10 min in the negatively charged (r)GO−COO− (pH 6.0), followed by three sequential rinsing steps in DI water for 1 min, and then dried with a gentle stream of nitrogen. The cationic (r)GO−NH_3_+ (pH 6) solution was subsequently deposited onto the (r)GO−COO− (pH 6.0) coated films using the same adsorption, washing sequence. The substrate was then placed into the OVA solution (1 mg/mL in 100 mM NaOAc buffer) for 10 min and exposed to the same rinsing steps as described above. Then, the graphene layer deposition procedure (GO−NH_3_+/(r)GO−COO) were repeated to deposit the first hexa-layer film (PAE/rGO/GO/OVA/GO/rGO)_n = 1_). These deposition processes were repeated until the desired numbers of hexa-layers (PAE/rGO/GO/OVA/GO/rGO)_n_ were coated on the substrate. The concentration of the carbon-object solutions used in all the deposition experiments was fixed to 0.1 wt% without any ionic salts. Film thickness was monitored by profilometry (Tecan) at five different predetermined locations on the dried film surface.

### Electrochemical Stimuli

A three-electrode system in a single compartment cell was used for electrochemical synthesis and characterization. The working electrode was gold-coated silicon (Au/Ti/silicon), and the counter electrode was platinum mesh. The reference electrode was Ag/AgCl. An EG&G (Model 273A) potentiostate/galvanostat was used for all electrochemical experiments.

### Calculation of protein release

Release experiments were conducted by immersing a prepared multilayer film into a 20 mL vial containing 3.0 mL of phosphate-buffered saline (PBS: water-based salt solution containing sodium chloride, sodium phosphate, potassium chloride, and potassium phosphate, the osmolarity and ion concentration of the solution usually similar with human body) at room temperature. At a series of different time points, films were transferred to another vial, and fresh PBS solution of same volume was introduced. Ova release from the multilayer film was followed by measuring the fluorescence spectra (Quantamaster Fluorometer, PTI) of released Fluorescein-labeled Ova in a PBS solution (absorption 494 nm/fluorescence emission 520 nm maxima). Calibration curve of fluorescence at certain predetermined concentration was prepared at every single released Ova measurement if fluorometer lamp had turned on. Every aliquots at certain time point of released OVA solution were applied to calibration curve to calculate exact amount of OVA. All release and film degradation studies were performed in triplicate.

In addition, we have performed circular dichroism (CD) spectroscopy of released media aliquots (by 0.5 V electric potential). The spectra shown here support that released ovalbumin is not denatured and retains its secondary structure (33% alpha-helix, 5% beta-structure, and 62% random coil) (supporting information S3).

## Results and Discussion

### Layer-by-Layer assembled multilayer film

**Figure 2:**
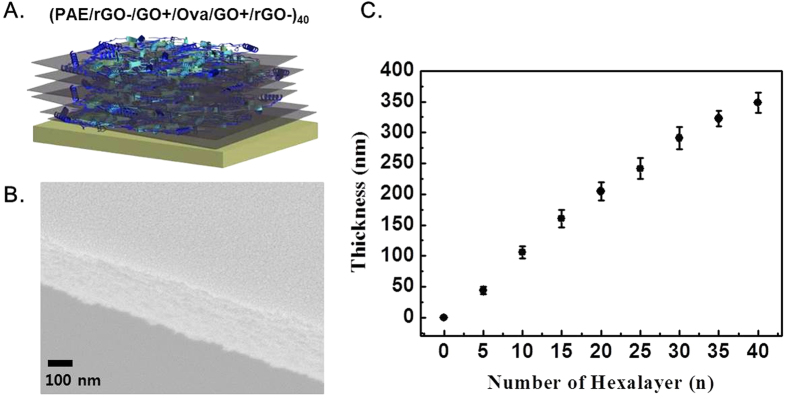
(**A**) Schematic illustration of the (PAE/rGO−/GO+/OVA/GO+/rGO−)_40_. (**B**) Representative surface morphology of a multilayer film: cross-sectional SEM image of as-assembled (PAE/rGO−/GO+/OVA/GO+/rGO−) _40_-multilayer films. Scale bar = 100 nm. (**C**) Growth curve for electrostatically assembled (PAE/rGO−/GO+/OVA/GO+/rGO−)_40_ multilayer films versus the number of hexalayers.

To fabricate an electro-responsive nanofilm capable of controlled ovalbumin (OVA) release, we engineered multilayer nanofilms with repeating hexalayer structures (PAE/rGO/GO/OVA/GO/rGO)_n_, *n = number of hexalayer*) by using poly(β-amino ester) (PAE), rGO, and GO as the building blocks (Fig. 2A). The major driving force for multilayer assembly is electrostatic interaction, where the adsorptions of the following occur in order onto the substrates: (i) positively charged PAE, (ii) GO and negatively charged OVA, and (iii) rGO^39^. The isoelectric point of OVA is 4.5^31^. As such, OVA were negatively charged at the experimental pH of 6 and 7.4 at which our nanofilms were deposited and tested, respectively. Here, we utilized PAE as a component of our multilayer films due to their hydrolytic degradation characteristics that enables the release of drugs from the films^38^. Scanning electron microscopy reveals the morphology of (PAE/rGO/GO/OVA/GO/rGO)_40_; although the multilayers yielded some crease features, we found that they were uniformly coated without defect and exhibited layered morphology (Fig. 2B). Under physiological conditions, the thickness of the multilayer nanofilms increases linearly with the increasing number of hexalayers (n) as shown in Fig. 2C, indicating that precise control of film thickness (or OVA loading) can be achieved using the designed hexalayer structures.

### Electrical stimuli and Protein release

**Figure 3:**
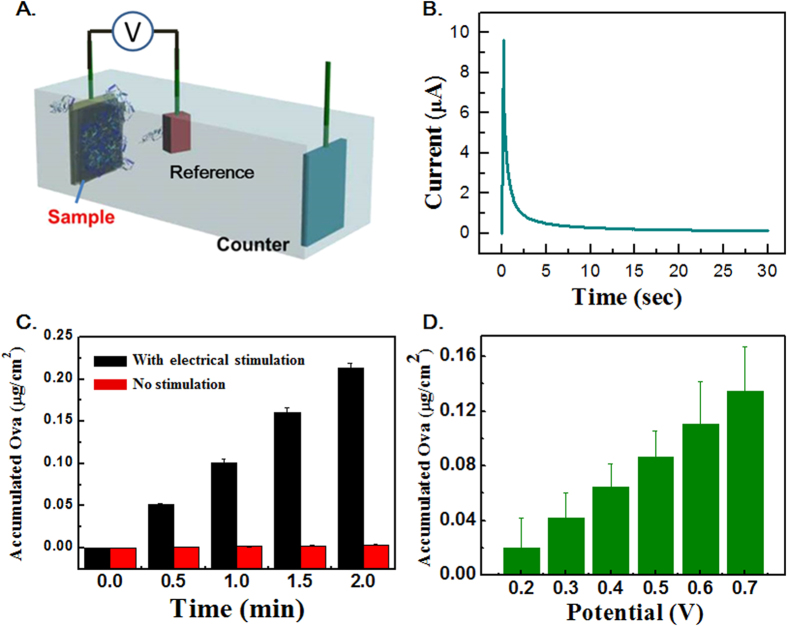
(**A**) Schematic illustration of the electrochemical setup for applying electrical stimuli. (**B**) Chronoamperometric response of the (PAE/rGO−/GO+/OVA/GO+/rGO−)_40_ film. A constant potential of 0.4 V is applied for 30 seconds. (**C**) (Red) Amount of protein released from (PAE/rGO−/GO+/OVA/GO+/rGO−)_40_ as a function of time when no electrical potential is applied. (Black) Amount of protein released from (PAE/rGO−/GO+/OVA/GO+/rGO−)_40_ as a function of time upon the application of 0.4 V. (**D**) Potential-dependent release of protein from (PAE/rGO−/GO+/OVA/GO+/rGO−)_40_ film.

Once it was established that our nanofilms could be built with a moderate control of film thickness (i.e. the amount of OVA), their protein release characteristics were examined after submerging them in phosphate-buffered solution (PBS) solution under physiological conditions (5% CO_2_, 37 °C). A schematic diagram of the three-electrode setup used for applying the electrical potential onto our nanofilms is shown in Fig. 3A. A constant potential ranging from 0.2–0.7 V was applied repeatedly at an interval of 30 minutes. Figure 3B shows a chronoamperogram (current vs. time) when a constant potential of 0.4 V (vs. Ag/AgCl) is applied onto the (PAE/rGO/GO/OVA/GO/rGO)_40_ film; an initial spike and then rapid decrease of current over time was observed, which is typical of a conductive thin film under the application of a constant potential. Based on the measured open circuit potential of about 0.1 V, the positive values of current (defined by the potentiostat/galvanostat used) indicate that hydrated anions and water move into the film to achieve surface charge neutrality when the positive potential of 0.4 V is applied.

Without electrical stimulation, our multilayer film, (PAE/rGO/GO/OVA/GO/rGO)_40_, released a negligible amount of OVA (<0.001 μg/cm^2^), with no sign of burst release for a few hours (Fig. 3C). These characteristics are consistent with previously reported GO− containing multilayer films^31^,^32^, indicating that our repeated hexalayer structures can also serve as barrier layers for the sustained release of therapeutic molecules. After confirming that the films do not release OVA abruptly without electrical stimulation, we tested the protein release characteristics of (PAE/rGO/GO/OVA/GO/rGO)_40_ when it is subject to stimulation.

Figure 3C shows the corresponding protein release profile, and clearly demonstrates that the nanofilms under electrical stimulation release about 50 times more OVA (0.05 μg/cm^2^) compared to no stimulation. This significant increase is due to the induction of charge imbalance on the surface of rGOx/GOx layers from the stimulation, and the subsequent hydrated anion and water influxes that compensate for this imbalance. We hypothesize that these influxes result in weakening of the electrostatic interactions (or crosslinks) between OVA and rGO layers. Eventually, hydrated anions replace the negatively charged OVAs, resulting in their release. Figure 3D shows the potential-dependent protein release data: higher potentials result in slightly more protein release, indicating more OVA replacement by anions during more positive electrical potential. In addition, upon applying a voltage, the (PAE/rGO−/GO+/OVA/GO+/rGO−)_40_ nanofilms exhibited a release of OVA and corresponded to a decrease in film thickness (supporting information S2). These data confirm that our nanofilms are not only capable of preventing the burst release of protein, but also capable of on-demand controlled release. These two features distinguish our LbL multilayer system from all the other previous LbL systems that suffer from either unwanted initial loss of protein or little real-time controllability of protein release^29^,^30^,^31^,^32^. Our concept of using the multilayer systems containing a barrier layer combined with an electrical stimulation could provide us a highly tunable protein delivery system to meet the complex, time-varying therapeutic needs that are typically required in various biomedical fields.

## Conclusion

In summary, we have successfully developed electro-responsive nanofilms that are capable of on-demand protein release. By introducing electrically conductive rGO layers into the multilayer structure of nanofilms, protein release could be easily adjusted by external electrical stimulation. Since the architecture and constituents of LbL assembly can be precisely adjusted, more elaborate release of protein can be achieved by manipulating both GO and rGO layers. Furthermore, the adaptable architecture of the nanofilm can act as a potential platform for the loading and release of numerous therapeutic protein molecules. Our strategy of using electrical stimulation with multilayer nanofilms can be extended for use in on-demand controlled release of molecules in various settings, including protein delivery, development of advanced biochips for drug screening, and biosensing.

## Additional Information

**How to cite this article**: Choi, M. *et al.* Multilayered Graphene Nano-Film for Controlled Protein Delivery by Desired Electro-Stimuli. *Sci. Rep.* **5**, 17631; doi: 10.1038/srep17631 (2015).

## Supplementary Material

Supplementary Information

## Figures and Tables

**Figure 1:**
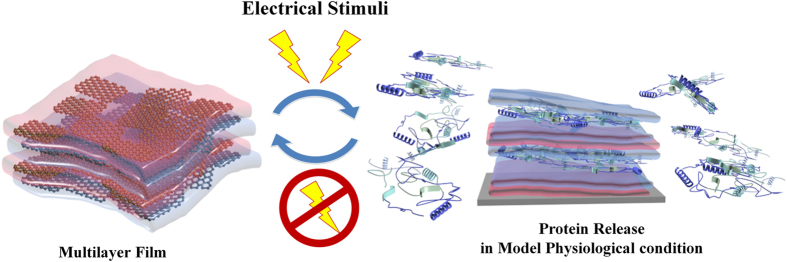
Schematic illustration of the manner by which protein is released from a multilayer film after electrical stimuli (The figure was drawn by M.C.).
