# Supplementary material for: Multilayered Graphene Nano-Film for Controlled Protein Delivery by Desired Electro-Stimuli
Source: Sci Rep. 2015 Dec 1;5:17631. doi: 10.1038/srep17631 (PMC4664934; doi:10.1038/srep17631)
Supplement: Supplementary Information [file srep17631-s1.doc]

Supporting Information:

Multilayered Graphene Nano-Film for Controlled
Protein Delivery by Desired Electro-Stimuli

**Moonhyun Choi^1^, Kyung-Geun Kim^2^, Jiwoong Heo^1^, Hyejoong Jeong^1^, Sung Yeol Kim^2,*^ and Jinkee Hong^1,*^**

^1^School of Chemical Engineering & Materials Science, Chung-Ang University,

Seoul 156-756, Republic of Korea

^2^School of Mechanical Engineering, Kyungpook National University,

Daegu 702-701, Republic of Korea
*Corresponding author: E-mail address: jkhong@cau.ac.kr, Tel.: +82-2-820-5561

Co-corresponding author: E-mail address: sykimknu@knu.ac.kr, Tel.: + 053-950-7595

**Figure S1**


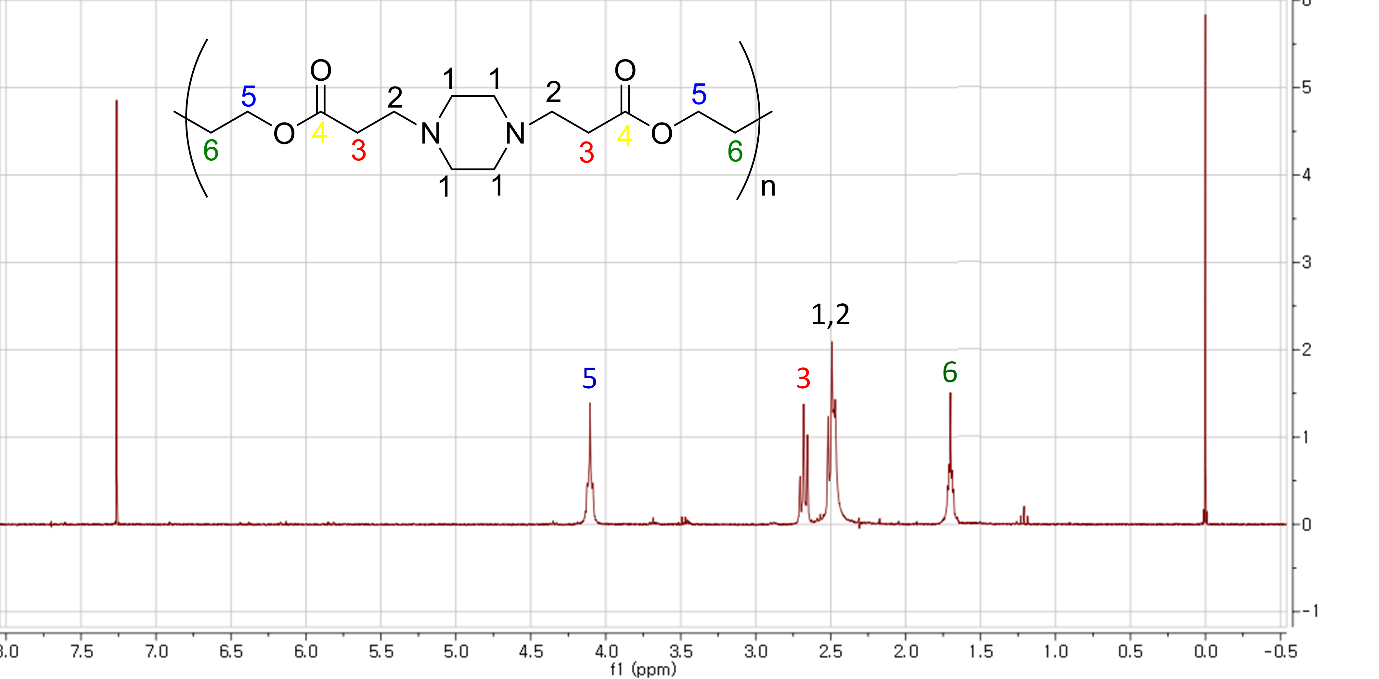


Figure S1. H-NMR data of PAE. ^1^H NMR *δ* (CDCl_3_, 300 MHz) 4.11 (br t, 4 H), 2.68 (br t, *J* = 7.55, 4 H), 2.49 (br m, 12 H), 1.71 (br m, 4H).

**Figure S2**

**
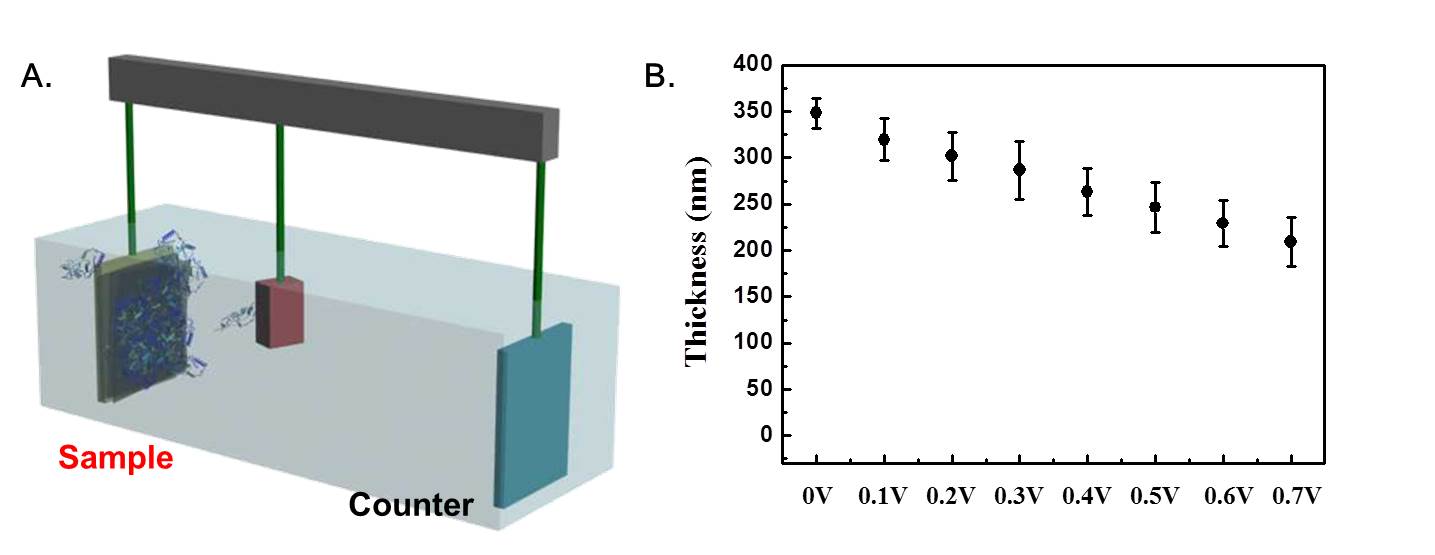
**

Figure S2. The change in film thickness for (PAE/rGO-/GO+/OVA/GO+/rGO-)_40_ as a function of applied voltage.

**Figure S3**

**
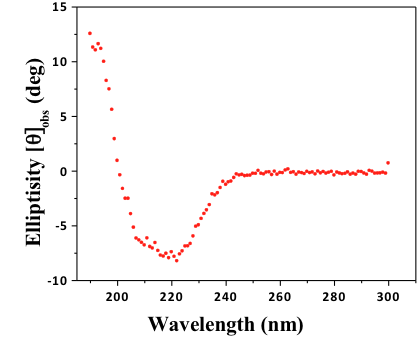
**

Figure S3. The CD spectra of Ovalbumin released by 0.5 V electric potential (conc. 0.08 μg/ml), and path length of the cuvette was 0.1 cm.
